# An overview on the protective effects of ellagic acid against heavy metals, drugs, and chemicals

**DOI:** 10.1002/fsn3.3704

**Published:** 2023-09-26

**Authors:** Zahra Jamshidi, Ali Roohbakhsh, Gholamreza Karimi

**Affiliations:** ^1^ Student Research Committee Mashhad University of Medical Sciences Mashhad Iran; ^2^ Department of Medicinal Chemistry, School of Pharmacy Mashhad University of Medical Sciences Mashhad Iran; ^3^ Pharmaceutical Research Center, Pharmaceutical Technology Institute Mashhad University of Medical Sciences Mashhad Iran; ^4^ Department of Pharmacodynamics and Toxicology, School of Pharmacy Mashhad University of Medical Sciences Mashhad Iran

**Keywords:** antioxidant, apoptosis, inflammation, oxidative stress, polyphenol, toxicity

## Abstract

Ellagic acid (EA) is a polyphenol extracted from many plants. EA modulates inflammatory mediators via antioxidant mechanisms, such as catalase (CAT) activities, superoxide dismutase (SOD), enhancement, increase in glutathione (GSH), and lipid peroxidation (LPO) suppression. EA has anti‐apoptotic properties that are thought to be mediated by regulating the expression of B‐cell lymphoma 2 (Bcl‐2), Bcl‐2‐associated X protein (Bax), and caspase‐3. In this article, we surveyed the literature dealing with the protective effects of EA against different heavy metals, drugs, and natural toxins. The findings indicated that EA has remarkable protective properties against various toxicants. Its protective effects were mostly mediated via normalizing lipid metabolism, oxidative stress, and inflammatory mediators, for example, tumor necrosis factor‐α (TNF‐α), interleukin‐6 (IL‐6), and IL‐1β. The results of this study showed that EA has significant protective effects against a varied range of compounds, either chemical or natural. These effects are mainly mediated via intensifying the antioxidant defense system. However, other mechanisms such as inhibition of inflammatory responses and suppression of apoptosis are important.

## Graphical abstract

Ellagic acid (EA) is a polyphenol extracted from many plants. EA modulates inflammatory mediators via antioxidant mechanisms. The findings indicated that EA has remarkable protective properties against various toxicants.

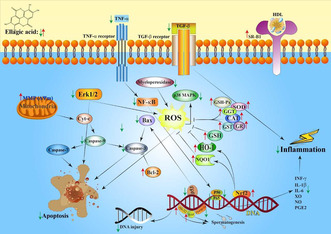

## 1 INTRODUCTION

**FIGURE 1:**
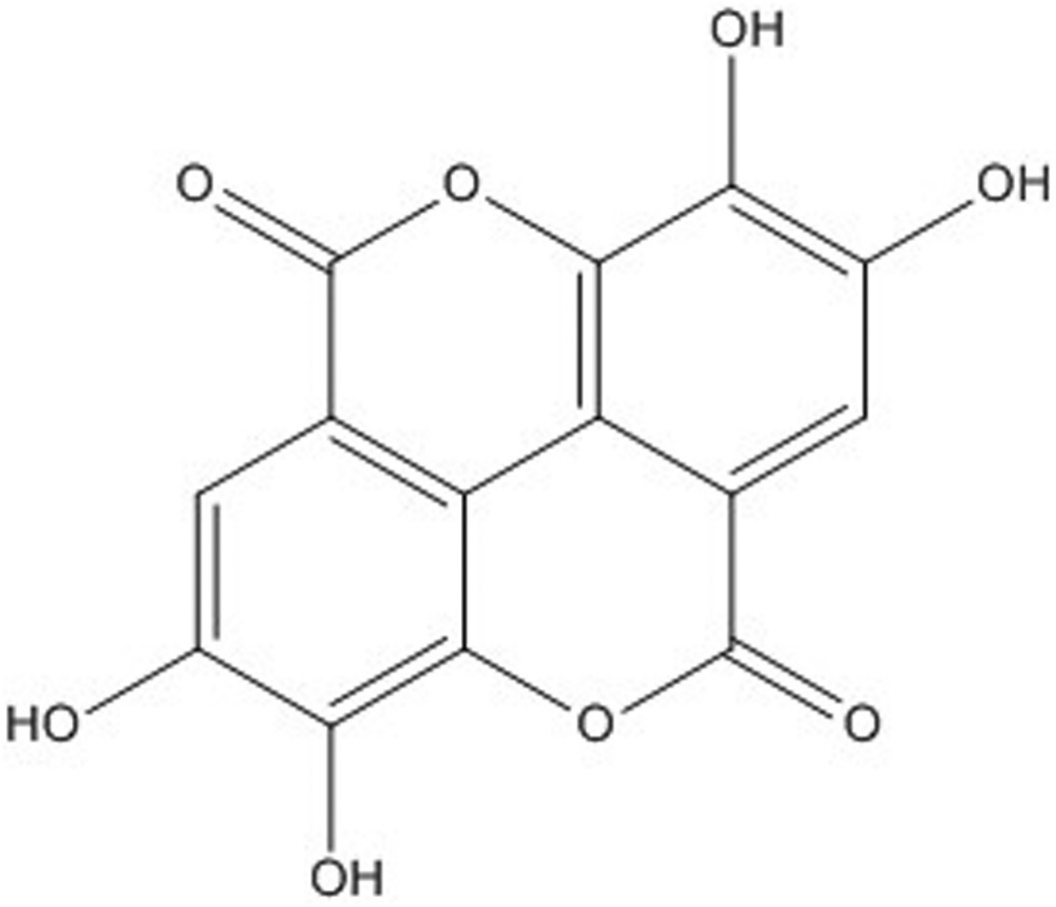
Chemical structure of ellagic acid (EA).

Human and animal tissues are regularly exposed to reactive oxygen species (ROS), including superoxide anion, hydrogen peroxide, hydroxyl radicals, and different radicals generated through numerous metabolic reactions (Hajam et al., 2022). The production of small amounts of free radicals appears to have an essential biological function, while oxidative stress results following extreme ROS production (Wu et al., 2020). Oxidative stress can be promoted by a diversity of factors such as exposure to drugs, heavy metals, and chemicals, (Possomato‐Vieira et al., 2018; Salazar‐Flores et al., 2020; Songbo et al., 2019; Yang et al., 2021). It may evoke serious interrelated derangements of main cellular structures and functions such as a change in the nucleic acid structure, alteration of proteins, disturbance in membrane ion transport and permeability, a raise in intracellular free calcium, and destruction of the cells by lipid peroxidation (Unsal et al., 2021). The human body has a collection of antioxidant defense mechanisms to control oxidative stress. Antioxidants including the non‐enzymatic [vitamin C, vitamin E, and glutathione (GSH)] and enzymatic [superoxide dismutase (SOD), catalase (CAT), and glutathione *S*‐transferase (GST)] antioxidants are known to decrease oxidative stress consequents by restoring abnormalities following exposure to chemicals and physical agents (Mishra & Srivastava, 2015). One of the critical components of the non‐enzymatic antioxidant defense is GSH. GSH is a vital substance in detoxification, nutrient metabolism, cell physiology, and regulation of cellular events containing DNA and protein synthesis, apoptosis and cell proliferation, gene expression, immune response, signal transduction, and cytokine production (Amin et al., 2021; Ghasemzadeh Rahbardar et al., 2021). Several natural polyphenol compounds were established to scavenge hydroxyl radicals and superoxide anions, block lipid peroxidation, and influence processes containing free radical‐mediated injury (Ahmadi et al., 2021; Naraki et al., 2021). Ellagic acid (EA, 2,3,7,8‐tetrahydroxychromeno[5,4,3‐*cde*]chromene‐5,10‐dione, Figure 1) is a polyphenol compound that is found in many plants and exhibits antioxidant (Bialonska et al., 2009) antianxiety (Kant et al., 2013), antiinflammatory (Promsong et al., 2015), antifibrogenic (Devipriya et al., 2007), antisteatosic (Yoshimura et al., 2013), and antiviral (Ajala et al., 2014; García‐Niño & Zazueta, 2015) properties.

**FIGURE 2:**
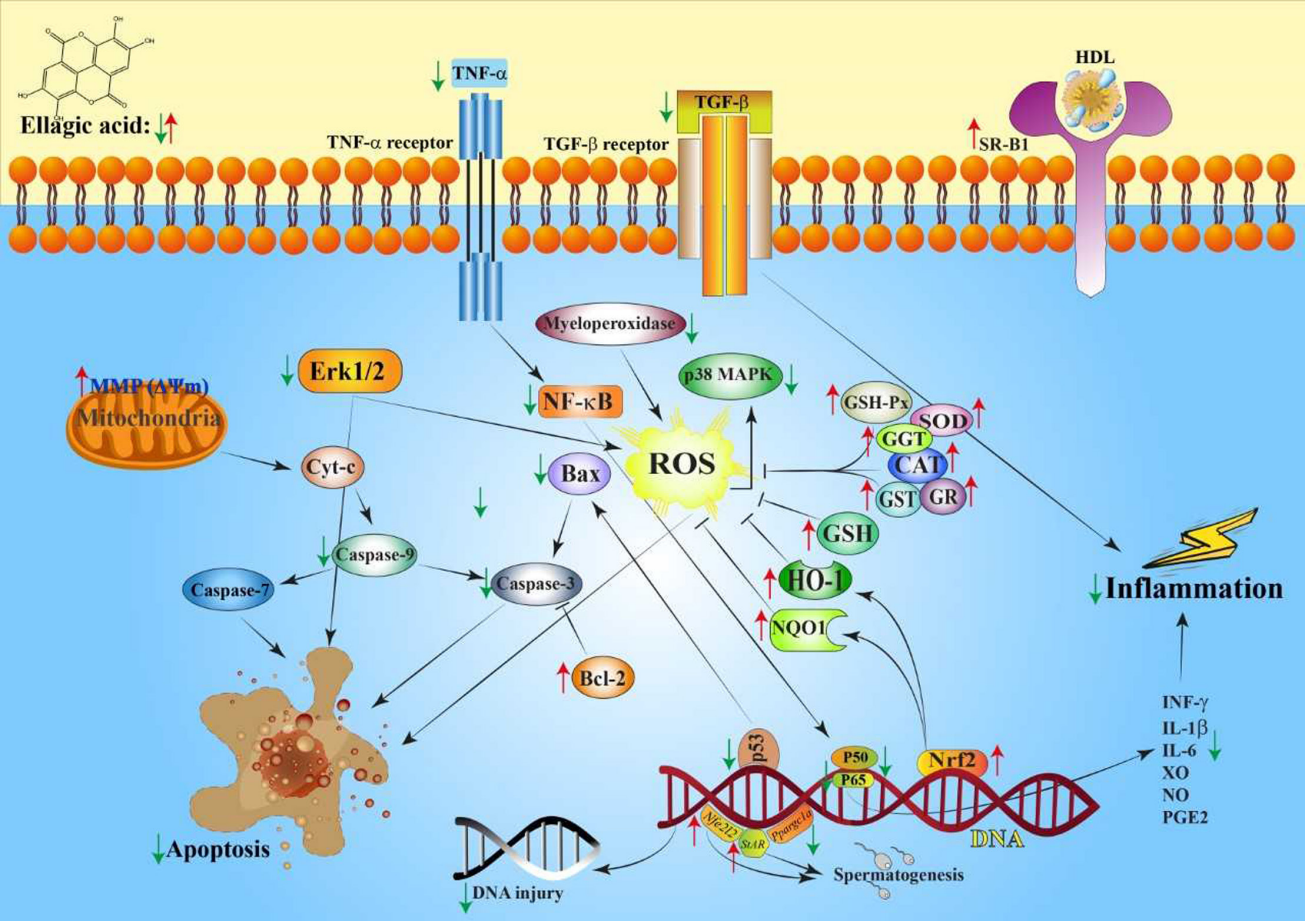
**S**chematic presentation of mechanisms displayed to mediate protective mechanisms of ellagic acid (EA). EA suppresses production of pro‐inflammatory and pro‐apoptotic mediators and boosts the antioxidant defense system. CAT, catalase; GSH, glutathione; IL‐1β, interleukine‐1β; IL‐6, interleukine‐6; *Nfe2l2*, nuclear factor erythroid 2‐like 2; ROS, reactive oxygen species; *StAR*, steroidogenic acute regulatory protein; SOD, super oxide dismutase; *Ppargc1a*, peroxisome proliferative activated receptor gamma coactivator 1alpha.

Various studies have exhibited that ellagic acid has the potential to reduce or prevent toxicity in the body via diverse mechanisms such as inhibiting nitric oxide (NO) generation, preventing nuclear factor‐kappaB (NF‐κB) activation, and intensifying the cellular antioxidant system (García‐Niño & Zazueta, 2015; Ríos et al., 2018). In the present review article, we summarize the EA protective effects against toxicities of heavy metals, drugs, and chemicals and discuss the mechanisms involved in EA protective actions (Figure 2).

## 2 METHOD

A comprehensive literature review was conducted using the following keywords: “ellagic acid” AND “protective” AND “toxicity” in the following databases: PubMed/Medline, Scopus, ScienceDirect, and Web of Science. Non‐English language, duplicated, and non‐relevant articles were excluded.

## 3 PROTECTIVE EFFECTS OF EA AGAINST HEAVY METALS TOXICITY

Heavy metal pollution is one of the main public health concerns worldwide. Heavy metals are not degradable and remain in the environment for several years, posing a main threat to human health (Anyanwu et al., 2018). The leading heavy metals with significant adverse effects are aluminum, lead, iron, arsenic, mercury, and cadmium. Heavy metal exposure can activate carcinogenic pathways. According to Kozlowski et al. (2014), toxicity depends on the type of ion and can affect specific tissues or, more broadly, the entire body can be poisoned (Kozlowski et al., 2014). Many studies have indicated that the mutagenicity and carcinogenicity of heavy metals are associated with the induction of oxidative stress (Fu & Xi, 2020; Genestra, 2007) that cause oxidative injury to proteins and DNA (Valko et al., 2006). Table 1 provides a brief of in vitro and in vivo studies concerning the protective effects of EA on selected heavy metals toxicities.

**TABLE 1:** Protective effect of ellagic acid against heavy metals toxicity.

Toxic agent	Dose/concentration of toxic agent, treatment period, and route of exposure	Dose/concentration of EA, treatment period, and route of administration	In vitro/In vivo model	Results of EA treatment	References
Arsenic	200 ppm, 40 days, oral	50 mg/kg, 40 days, oral	Male Swiss mice	Regulating the expression of *Ppargc1a, Nfe2l2*, and *StAR* genes	Guvvala et al. (2019)
	5 mg/kg, 10 days, intraperitoneal	30 mg/kg, 10 days, oral	Male Wistar rats	Reduced lipid peroxidation	Hemmati et al. (2018)
	10 mg/kg, 21 days, oral	30 mg/kg, 14 days, oral	Male Wistar rats	Decreased both the MDA and NO levels and enhanced CAT, SOD, and GPx activities	Mehrzadi et al. (2018)
	20, 40, and 100 μM, 1 h	20, 40, and 80 μM, 1 h	Isolated rat mitochondria	Reduced mitochondrial damage, ROS, restored total dehydrogenase activity	Keshtzar et al. (2016)
	10 mg/kg, 21 days oral	10 and 30 mg/kg, 21 days	Male Wistar rats	Increased GPx, SOD, CAT activities and GSH level and decreased NO and MDA levels	Mehrzadi et al. (2018)
	10 mg/kg, 8 days, oral	20 and 40 mg/kg, 11 days, oral	Wistar rats	Decreased ROS Bax, Bcl‐2, IL‐1β, TNF‐α, caspase 3, and IFN‐γ	Firdaus et al. (2018)
Chromium	100 μM, 24 h	25, 50, and 100 μM, 1 h	Primary human lymphocytes	Enhanced IL‐2 and decreased the number of apoptotic cells	Bodiga et al. (2022)
Mercuric	1.23 mg/kg, 7 days, intraperitoneal	5 mg/kg, 7 days, oral	Male rats	Increased GSH, GPx, CAT, and SOD	Bharathi and Jagadeesan (2014)
	1.23 mg/kg, 7 days, intraperitoneal	5 mg/kg, 7 days, oral	Male rats	Reduced ALT, ALP, AST, LDH, bilirubin, cholesterol urea, and albumin, creatinine levels in the serum	Jagadeesan and Bharathi (2014)
Cobalt	100 μM, 24 h	25, 50, and 100 μM, 1 h	Primary human lymphocytes	Improved the amount of IFN‐γ secreted	Bodiga et al. (2022)
Lead	100 mg/kg, 8 weeks, oral	300 and 500 mg/kg, 8 weeks, oral	Laying Japanese quails	Decreased caspase‐3 and caspase‐9 and MDA level and increased, GSH‐Px, and CAT	Iflazoglu Mutlu et al. (2021)

Abbreviations: ALP, alkaline phosphatase; ALT, alanine aminotransferase; AST, aspartate aminotransferase; Bax, Bcl‐2‐associated X protein; Bcl‐2, B‐cell lymphoma 2; CAT, catalase; GPx, glutathione peroxidase; GSH‐Px, plasma glutathione peroxidase; IFN‐γ, interferon‐γ; IL‐1β, Interleukin‐1 beta; IL‐2, interleukin‐2; MDA, malondialdehyde; *Nfe2l2*, nuclear factor erythroid 2‐ like 2; NO, nitric oxide; *Ppargc1a*, peroxisome proliferative activated receptor gamma coactivator 1alpha; ROS, reactive oxygen species; SOD, superoxide dismutase; *StAR*, steroidogenic acute regulatory protein; TNF‐α, tumor necrosis factor‐α.

### 3.1 Arsenic

Arsenic contamination is a serious worldwide health risk to the human population due to its carcinogenic nature and highly toxic properties (Mawia et al., 2021). Arsenic levels in the environment are increasing and threaten ecosystems through both natural and anthropogenic activities. The international agency for research on cancer categorizes arsenic in group 1, carcinogenic to humans (Khairul et al., 2017). Binding to thiols, oxidative stress induction, and methylation have been proposed as potential mechanisms for arsenic toxicity in the endocrine system (Nurchi et al., 2020). Oxidative stress is a major cause of male infertility following arsenic‐induced testicular toxicity. Arsenic, via free radical‐mediated reactions, evokes DNA injury in sperm cells (Flora & Agrawal, 2017). A recent study reported that EA significantly restored CAT enzyme activity, total antioxidant capacity) TAC(, and GSH levels, and reduced malondialdehyde )MDA(. Besides, EA increased sperm quality, restored antioxidant balance, and regulated the expression of nuclear factor erythroid 2‐like 2 (*Nfe2l2*), peroxisome proliferative activated receptor gamma coactivator 1alpha (*Ppargc1a*), and steroidogenic acute regulatory protein (StAR) genes in the testis (Guvvala et al., 2019). *Nfe2l2* is a gene involved in the expression of antioxidant proteins that protect against inflammation and oxidative damage. It serves as a biomarker for the prediction of ROS‐linked male infertility (Chen et al., 2012). *Ppargc1a* is a stress response gene upregulated by cellular stress (Rius‐Pérez et al., 2020). Arsenic down‐regulates *StAR* signaling and production of steroid hormones and promotes oxidative stress that leads to reproductive health impairment in males (Kadry & Megeed, 2018). Another study indicated that EA decreased caspase‐3 activity and, interleukin‐1 beta (IL‐1β), tumor necrosis factor‐α (TNF α), and Bcl‐2‐associated X protein (BAX) along with upregulation of B‐cell lymphoma 2 (Bcl‐2) expression in arsenic exposed rats (Firdaus et al., 2018). Mehrzadi et al. reported that by increasing serum testosterone and testicular antioxidant markers, EA improved arsenic‐induced testicular toxicity. EA treatment decreased MDA, NO, TNF‐α, and IL‐1β significantly and increased glutathione peroxidase (GPx), SOD, CAT, and GSH content (Table 1; Mehrzadi et al., 2018). In summary, EA by suppressing oxidative stress, activating *Nfe2l2* and *StAR* gene expressions, and suppressing the *Ppargc1a* pathway protected against arsenic testicular toxicity.

### 3.2 Chromium

Chromium is the most abundant mineral in the Earth's crust. Contaminated well water is considered the major route of chromium exposure in humans. Toxic effects include mouth ulcers, indigestion, vomiting, acute tubular necrosis, abdominal pain, kidney failure, and even death (Pavesi & Moreira, 2020). It was shown that EA significantly protected against chromium‐induced toxicity in primary human lymphocytes. Besides, EA decreased the apoptotic index and improved the viability and proliferative responses of both activated and resting lymphocytes, and normalized the cytokine secretion such as interleukin‐2 (IL‐2) and interferon‐γ (IFN‐γ) from the activated lymphocytes (Table 1; Bodiga et al., 2022).

### 3.3 Mercury

Mercury is a toxic element that has raised worldwide public health concerns due to its toxicity. Anxiety, sleep disturbance, and depression are psychological problems attributed to acute exposure to mercury (Raj & Maiti, 2019). Oxidative stress is a fundamental mechanism for mercury‐induced liver injury. Treatment of rats with EA after mercury exposure remarkably elevated antioxidant enzymes' activities such as GPx, SOD, and CAT. Furthermore, EA decreased the oxidant content and simultaneously intensified the antioxidant system in the kidney tissue. Mercury‐intoxicated rats treated with EA showed no swelling and disorientation in the hepatocytes (Table 1; Bharathi & Jagadeesan, 2014; Jagadeesan & Bharathi, 2014).

### 3.4 Cobalt

Cobalt is a widely dispersed pollutant with many adverse health effects. In humans, systemic cobalt toxicity manifests as a clinical syndrome with a different presentation of neurological, endocrine, and cardiovascular symptoms (Leyssens et al., 2017). EA promoted potent cytoprotective and antigenotoxic effects against cobalt‐induced toxicity in primary human lymphocytes by increasing IL‐2 and IFN‐γ levels. Pretreatment with EA even in the presence of metal ions effectively enhanced the IL‐2 level secreted by anti‐CD3‐activated lymphocytes. It also modified the amount of IFN‐γ secreted by the activated lymphocytes even in the presence of cobalt and reduced the number of apoptotic cells (Table 1; Bodiga et al., 2022).

### 3.5 Lead

Lead is a heavy metal and an environmental pollutant that is toxic even in very low concentrations. Lead causes ROS production, liver injury, DNA damage, oxidative stress, and apoptosis. It was observed that EA administration to lead‐treated laying Japanese quails reduced serum MDA levels while increasing plasma glutathione peroxidase (GSH‐Px), and CAT values. In addition, EA treatment remarkably down‐regulated caspase‐3 and ‐9 levels in liver tissue. Laying quails fed with EA showed enhanced egg production and weight (Table 1; Iflazoglu Mutlu et al., 2021).

## 4 PROTECTIVE EFFECTS OF EA AGAINST DRUGS TOXICITIES

**FIGURE 3:**
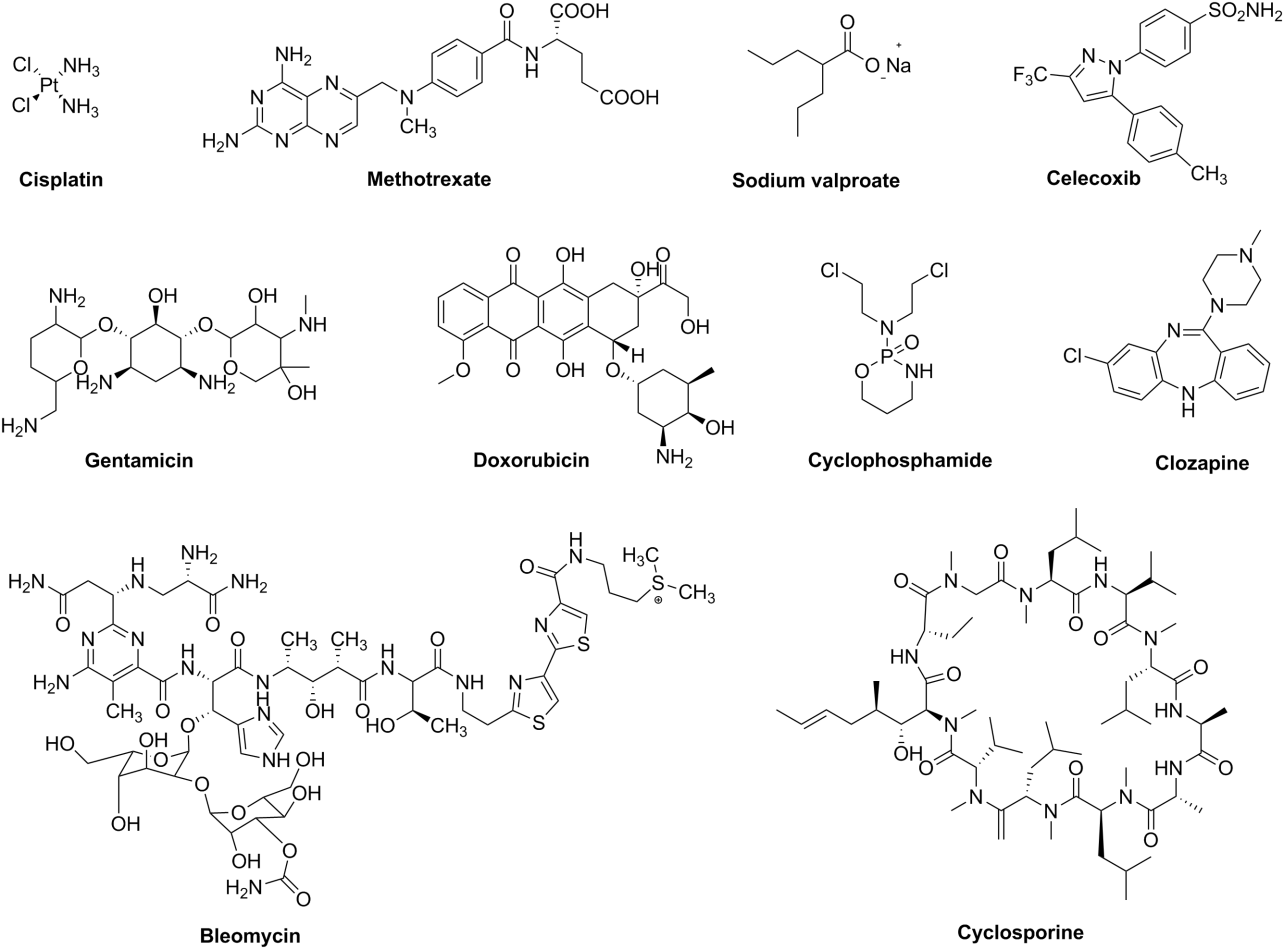
Chemical structure of drugs whose toxicity is reduced by ellagic acid (EA).

The greatest problem during the development of new drugs is organ toxicity (Lin & Will, 2012). Two important reasons for drug attrition are hepatotoxicity and cardiotoxicity (Schuster et al., 2005). Table 2 provides a brief of in vitro and in vivo studies on the effects of EA on selected drug toxicities. The chemical structure of drugs whose toxicity is reduced by EA is shown in Figure 3.

**TABLE 2:** Protective effects of ellagic acid (EA) against drugs toxicities.

Toxic drug	Dose/concentration of toxic drug, treatment period, and route of exposure	Dose/concentration of EA, treatment period, and route of administration	In vitro/In vivo model	Results of EA treatment	References
Cisplatin	5 mg/kg, once in a week for 4 weeks, intraperitoneal	10 mg/kg, 6 weeks, oral	Male Laca mice	Reduced LPO and increased GSH levels	Goyal et al. (2019)
	7 mg/kg, 10 days, intraperitoneal	10 mg/kg, 10 days, oral	Male Sprague–Dawley rats	Reduced MDA level, increased GSH‐Px, GSH, and CAT	Yüce et al. (2007)
	5 mg/kg, once a week for 4 weeks, intraperitoneal	10 mg/kg, daily for 6 weeks, oral	Male Laca mice	Reduced LPO and increased GSH levels	Goyal et al. (2019)
Bleomycin	10 U/kg, single dose, intrathecal	15 mg/kg, 14 days, oral	Male Wistar rats	Reduced MPO, NO, LPO, and lung hydroxyproline level and increased GSH	Saba Khan et al. (2013)
Cyclophosphamide	150 mg/kg, intraperitoneal	15 mg/kg, 14 days, oral	Male Wistar rats	Reduced MPO, NO, LPO, and lung hydroxyproline level and increased GSH	Saba Khan et al. (2013)
	15 mg/ kg, once a week for 8 weeks, oral	2 mg/kg, every other day for 8 weeks, oral	Male Sprague–Dawley rats	Decreased MDA and increased GSH and CAT	Ceribaşi et al. (2010)
	50 mg/kg, Single dose, intraperitoneal	50 and 100 mg/kg, 1 week, oral	Male Swiss albino mice	Increased GSH, GR, GST, GPx, and decreased XO, MDA, and GGT	Rehman et al. (2012)
Celecoxib	16 μg/mL, 6 h	10, 50, and 100 μM, 6 h	Isolated rat heart cardiomyocytes and mitochondria	Decreased the MDA, mitochondrial swelling, ROS formation, GSSG, and increased GSH	Atashbar et al. (2021)
Cyclosporine	15 mg/kg, 30 days, oral	10 mg/kg, 30 days, subcutaneous injection	Albino rats	Decreased MDA levels and ameliorated GSH, CAT	Abdul‐Hamid et al. (2016)
	25 mg/kg, 21 days, oral	12.5, 25, and 50 mg/kg, 21 days, oral	Male albino Wistar rats	Decreased AST, ALT, ALP, and LDH, TBARS and increased SOD, CAT, GST, vitamin C, vitamin E, and GSH	Pari and Sivasankari (2008)
Gentamicin	100 mg/kg, 10 days, intraperitoneal	10 mg/kg, daily for 10 days, oral	Male Sprague–Dawley rats	Normalized MDA, MMP levels and mitochondrial swelling and decrease ROS formation and increased GSH, CAT, SOD activity	Sepand et al. (2016)
Sodium valproate	400 mg/kg, 7 days, oral	10, 25, 50 mg/kg, 1 week, oral	Male Wistar rats	Increased body weight of rats, weights of the testes, and sperm count and decrease sperm abnormalities	Girish et al. (2014)
Doxorubicin	20 mg/kg, 72 h, intraperitoneal	0.25, 0.5, and 1 g, 8 weeks, oral	Male C57BL/6 mice	Suppressed MAPK pathways and NF‐κB and decreased ROS, inflammatory cytokines, and cleaved caspase‐3	Lin and Yin (2013)
	5 mg/kg, twice a week for 2 weeks, intraperitoneal	10 mg/kg, 14 days, oral	Male Sprague–Dawley rats	Normalized MDA and GSH testicular contents and reduced the TNF‐α level	Georgy and Maher (2017)
	2 mg/kg, once a week for 8 weeks, intraperitoneal	2 mg/kg, every other day for 8 weeks, oral	Male Sprague–Dawley rats	Reduced MDA and increase in GSH levels, GSH‐Px, CAT activity and the plasma testosterone levels	Çeribaşı et al. (2012)
Methotrexate	20 mg/kg, 5 days, intraperitoneal	10 mg/kg, 5 days, oral	Male Wistar rats	Decreased serum levels of PGE2, MDA, NO, MPO, XO, and AD and increased GSH	El‐Boghdady (2011)
Bevacizumab	50 and 100 μg/mL, 1 h	10–100 μM, 1 h	Rat heart mitochondria	Decreased ROS formation	Mohammad Khanlou et al. (2022)
Clozapine	50 μM, 4 h	10, 20, and 50 μM, 4 h	Isolated cardiomyocytes	Reduced MDA, and GSSG, and increased the GSH	Ahangari et al. (2022)

Abbreviations: AD, adenosine deaminase; ALP, Alkaline phosphatase; ALT, Alanine aminotransferase; AST, aspartate aminotransferase; CAT, catalase; GGT, γ‐glutamyltransferase; GPx, glutathione peroxidase; GR, glutathione reductase; GSH, glutathione; GSH‐Px, plasma glutathione peroxidase; GSSG, glutathione disulfide; GST, glutathione‐*S*‐transferase; LDH, lactic dehydrogenase; LPO, lipid peroxidation; MDA, malondialdehyde; MMP, mitochondrial membrane potential; MPO, myeloperoxidase; NF‐κB, nuclear factor‐kappaB; NO, nitric oxide; PGE2, Prostaglandin E2; ROS, reactive oxygen species; TBARS, thiobarbituric acid reactive substances; TNF‐α, tumor necrosis factor alpha; XO, xanthine oxidase.

### 4.1 Cisplatin

Cisplatin is a commonly used chemotherapeutic agent for various tumors. Nephrotoxicity is a common toxicity of cisplatin as renal tubule cells are sensitive to cisplatin accumulation (Motwani et al., 2022). A study showed that the antioxidant property of EA elicited beneficial effects on cisplatin‐induced nephrotoxicity and gonadotoxicity. Cisplatin caused enhancement in lipid peroxidation (LPO), and ROS, and diminished GSH content in the kidney and testis. These effects were reversed by EA administration in rats (Goyal et al., 2019). Cisplatin also caused oxidative damage in the liver and heart tissues via overproduction of free radicals. EA significantly reduced MDA levels and improved GSH‐Px, GSH, and CAT in the heart and liver tissues of cisplatin‐treated rats. Decreased lipid peroxidation in these tissues indicates that EA efficiently suppressed oxidative DNA damage and scavenged free radicals (Table 2; Yüce et al., 2007).

### 4.2 Bleomycin

Chemotherapeutic drugs treat a diverse range of tumors such as the kidney, lung, colon, and pancreas. To avoid drug resistance and enhancement the chemotherapeutic efficacy, combination therapy is used (Leary et al., 2018; Zhan & Wang, 2018). Bleomycin and cyclophosphamide, two anticancer drugs, are used in combination to treat squamous cell carcinoma of the head and neck. Combination therapy has frequently been associated with pulmonary toxicity (Méndez‐Echevarría et al., 2018). Lipid peroxides are key signal transduction mediators for inflammatory modifications in lung tissue. Bleomycin and cyclophosphamide administration increased oxidative stress by increasing LPO and protein oxidation (Saba Khan et al., 2013). EA treatment in rats exposed to bleomycin and cyclophosphamide showed reduced myeloperoxidase (MPO), NO, LPO, and hydroxyproline in lung tissues. Also, histopathological studies provided evidence for the protective effect of EA on bleomycin and cyclophosphamide‐induced pulmonary toxicity in rats (Table 2; Saba Khan et al., 2013).

### 4.3 Cyclophosphamide

Cyclophosphamide is an orally active alkylating agent that is used widely as an anticancer agent (Emadi et al., 2009). Side effects associated with cyclophosphamide use include bone marrow toxicity, infections, hemorrhagic cystitis, gastrointestinal abnormalities, and hair loss (Ponticelli & Glassock, 2019). One of the toxic consequences of cyclophosphamide administration is increased lipid peroxidation, and GSH‐Px, GSH, CAT, and SOD reduction in the testis (Ghosh et al., 2002; Selvakumar et al., 2006). In a study, cyclophosphamide administration caused fundamental raises in total abnormality of sperm and tail, plasma MDA level and erythrocyte SOD activity and reduced erythrocyte CAT activity, germinal cell layer thickness, diameters of seminiferous tubules, and Johnsen's Testicular Score along with degeneration, immature germ cells, necrosis, congestion and atrophy in testicular tissue of the rats. EA treatments reduced cyclophosphamide‐induced lipid peroxidation and normalized testicular histopathology and sperm morphology (Ceribaşi et al., 2010). Rehman et al. using cyclophosphamide‐induced kidney and testicular toxicities showed that EA increased GSH, GR, GST, and GPx levels but decreased xanthine oxidase (XO), MDA, and γ‐glutamyltransferase (GGT) levels. In addition, cyclophosphamide caused renal damage that was characterized by an increase in BUN, LDH, and creatinine levels, and EA prevented cyclophosphamide‐induced renal toxicity by a decrease in serum BUN, creatinine, and lactic dehydrogenase (LDH) (Table 2; Rehman et al., 2012).

### 4.4 Celecoxib

The first member of the nonsteroidal anti‐inflammatory drugs (NSAIDs) that preferentially inhibits the cyclooxygenase‐2 (COX‐2) enzyme is celecoxib. COX‐2 enzyme inhibitors were developed to treat arthritis pain and inflammation without the adverse side effects associated with previously accessible NSAIDs. Celecoxib may cause serious cardiovascular problems, including congestive heart failure, myocardial infarction, and stroke (Cheng et al., 2021). Atashbar et al. found that EA treatment significantly reduced ROS formation and glutathione disulfide (GSSG) levels initially increased due to celecoxib administration in cardiomyocytes. EA also prevented celecoxib‐induced mitochondrial membrane potential (MMP) collapse (Atashbar et al., 2021).

### 4.5 Cyclosporine

Cyclosporine A is a neutral lipophilic cyclic undecapeptide with potent immunosuppressive properties (Wu et al., 2018). The potent immunosuppressant effect of cyclosporine A is due to its specific blocking effect on lymphokine generation, differentiation, and signal transduction pathways of T cells. Therefore, it prevents cytokine production and suppresses immune response. On the other hand, cyclosporine A activates the pro‐oxidant pathways and raises the synthesis of ROS and lipid peroxidation products (Rezzani, 2006). In an experimental study, administration of EA to cyclosporine A‐treated rats significantly reduced MDA levels and ameliorated GSH, CAT, and peroxidase (Px) (Abdul‐Hamid et al., 2016). Rats exposed to cyclosporine A showed increases in the expression of hepatic markers for example aspartate aminotransferase (AST), alanine aminotransferase (ALT), alkaline phosphatase (ALP), and lactic dehydrogenase (LDH) whereas, EA treatment decreased hepatic markers, and increased tissue enzymatic (CAT, SOD, and GST) and non‐enzymatic (vitamin E, vitamin C, and GSH) antioxidants (Table 2; Pari & Sivasankari, 2008).

### 4.6 Gentamicin

Gentamicin belongs to the aminoglycoside family of antibacterial drugs used against gram‐negative aerobic bacterial infections. Long‐term clinical application of gentamicin may result in nephrotoxicity and ototoxicity (Brkić et al., 2022). It was reported that EA remarkably ameliorated SOD and CAT enzyme activity, and GSH in kidney tissue. EA protected against gentamicin‐induced mitochondrial damages as demonstrated by lowering mitochondrial ROS content, preventing MMP loss, decreasing mitochondrial swelling, and reducing cytochrome c release. An increase in Bcl‐2/Bax expression and caspase‐3 activation observed in the gentamicin‐treated group was remarkably ameliorated by EA treatment (Table 2; Sepand et al., 2016).

### 4.7 Sodium valproate

Sodium valproate is an antiepileptic drug with a wide range of applications. It is used in other neurological disorders conditions, such as migraine, schizophrenia, depression, and bipolar disorder, as well (Nanau & Neuman, 2013). Research on animal models demonstrated that valproate causes reversible modification in the sperm morphology, sperm motility, sperm count, and histoarchitecture of the testes. Girish et al. reported that oral administration of EA attenuated valproate‐induced testicular and spermatozoal damages. In that study, EA showed a protective effect against valproate‐induced weight gain, lower sperm count, and sperm abnormalities (Table 2; Girish et al., 2014). This action of EA may be related to its antiapoptotic and antioxidant properties.

### 4.8 Doxorubicin

Doxorubicin is a key anticancer drug for solid and hematologic tumors. However, clinical application of doxorubicin has been associated with significant cardiotoxicity. Cardiotoxicity is mediated via enhanced oxidative stress, caspase‐3, NF‐κB, and mitogen‐activated protein kinase (MAPK) activity (Tacar et al., 2013). In a rat model of doxorubicin‐induced cardiotoxicity, pretreatment with EA reduced caspase‐3, NF‐κB p50, NF‐κB p65, phospho‐p38 (p‐p38), and phospho‐extracellular signal‐regulated kinases 1/2 (p‐ERK1/2) levels. EA pretreatment remarkably reduced plasma C‐reactive protein (CRP) and cardiac IL‐10, monocyte chemoattractant protein‐1 (MCP‐1), TNF‐α, and IL‐6 levels. EA also decreased ROS, MDA, and XO, and increased GPx and SOD activities in the heart (Lin & Yin, 2013). In another study, pretreatment with EA significantly restored the normal testicular relative weight, improved spermatological impairment, normalized both GSH and MDA testicular contents, and reduced the TNF‐α level (Georgy & Maher, 2017). In doxorubicin‐induced testicular toxicity, EA increased tissue GSH level, CAT and GSH‐Px activities, and plasma testosterone level (Table 2; Çeribaşı et al., 2012). These outcomes suggest that EA has protective effects against doxorubicin‐induced apoptosis, inflammation, oxidative stress, and ameliorates expressions of MCP‐1, TNF‐α, IL‐6, and IL‐10. Furthermore, EA via induction of endogenous antioxidant enzymes, by phosphorylation of ERK1/2, prevented toxicity in cardiomyocytes.

### 4.9 Methotrexate

Methotrexate is a folate analog that inhibits RNA and DNA synthesis (Singh et al., 2012). Mastitis, nausea, vomiting, liver enzyme elevation, bone marrow suppression, hepatotoxicity, and multiorgan toxicity are common side effects of methotrexate (Howard et al., 2016). In a rat model of methotrexate‐induced small intestine damage, animals pretreated with EA exhibited a reduction in serum levels of prostaglandin E2 (PGE_2_), MDA, NO, MPO, XO, and adenosine deaminase (AD) activities, increased GSH levels, and modified intestine morphology (Table 2; El‐Boghdady, 2011).

### 4.10 Bevacizumab

Bevacizumab is an antiangiogenic drug that has been approved for the treatment of various cancers. Bevacizumab inhibits vascular endothelial growth factor (VEGF) and hypoxia‐inducible factor signaling (HIF) (de Aguiar & de Moraes, 2019). Two percent of patients treated with bevacizumab show cardiomyopathy and heart failure (Bordun et al., 2015). In another study, the enzymatic activities of oxidative phosphorylation complexes I, II, III, I + III, IV, II + III, and citrate synthase were evaluated in isolated rat heart intact mitochondria in the presence of bevacizumab. Complex II activity was remarkably decreased compared with the control group and activities of other complexes were not affected by bevacizumab. This conclusion confirmed that complex II is the principal oxidative phosphorylation target for bevacizumab in rat heart mitochondria. Bevacizumab causes dysfunction in the rat heart mitochondria by complex II inhibition, causing permeabilizing of the mitochondrial membrane and mitochondrial swelling. The study showed protective of EA remarkably elevated complex II activity and stopped mitochondrial swelling compared to treated groups with bevacizumab alone (Mohammad Khanlou et al., 2022).

### 4.11 Clozapine

Clozapine is a tricyclic dibenzodiazepine used to treat resistant schizophrenia. Adverse reactions associated with clozapine are hypotension, blood dyscrasia, neutropenia, agranulocytosis, sedation, seizures, metabolic syndrome, constipation, cardiotoxicity, and hypersalivation (De Fazio et al., 2015). Treatment of rats with EA reduced clozapine‐induced ROS formation, decreased GSSG and MDA, and increased GSH. The findings implied that EA protected cardiomyocytes from oxidative injury by inhibiting mitochondrial dysfunction, ROS formation, and lysosomal damage (Ahangari et al., 2022).

## 5 EA AGAINST CHEMICALS TOXICITIES

Humans and animals are exposed to diverse synthetic chemicals and natural substances through the environment. Above 140 million chemical substances have been registered by Chemical Abstract Service (CAS) as of June 2018 (Tang et al., 2018). A chemical can cause acute and chronic toxicities depending the conditions of exposure. A brief of in vitro and in vivo studies that examined the effects of EA against chemical toxins toxicities is given in Table 3.

**TABLE 3:** Protective effects of ellagic acid (EA) against chemicals toxicity.

Toxic chemical	Dose/concentration of toxic chemical, treatment period, and route of exposure	Dose/concentration of EA, treatment period, and route of administration	In vitro/In vivo model	Results of EA treatment	References
Paraquat	15 and 45 mg/kg, 1 hour, intraperitoneal	85 mg/kg, single dose, 24 hours, oral	Female Wistar rats	Decreased TOS and increased TAS levels	Silfeler et al. (2017)
Ethanol	100 mM, 24 h	1, 10, and 100 μM, 2 h	Human hepatocellular carcinoma (HepG2) cells	Decreased TGF‐β1, NO and increased the expression of SR‐B1	Sohn et al. (2013)
	7.9 g/kg, 45 days, oral	30, 60, and 90 mg/kg, 45 days, intragastric intubation	Female Wistar rats	Lowered the liver marker enzymes (AST and ALT), and lipid peroxidative markers (TBARS and HP), and modulated the lipid metabolism	Devipriya et al. (2008)
Phosalone	0.01, 0.1, 1 and 10 mM, 24 h	1, 10, 100, and 1000 nM, 24 h	Rat embryonic fibroblast cells	Decreased inflammatory markers)TNF‐α, IL‐1β, IL‐6β) and reduced the expression of NF‐κB, RB and p53	Baeeri et al. (2017)
Rotenone	0.1 μM, 24 h/1 mg/kg, 6 times a week for consecutive 5 weeks, subcutaneous injection	0.1 and 1 μM, 30 min/100 and 20 mg/kg, once a day for 5 weeks, intragastric	MN9D, BV2 and C6 / homozygous Nrf2 knockout male mice and Wild‐type C57BL/6J male mice and	Activated Nrf2 signaling pathway and elevated Nrf2, NQO1, and HO‐1 expressions	Wei et al. (2020)
Acrylamide	20 mg/kg, 30 days, oral	10 and 30 mg/kg, 30 days, oral	Male Wistar rats	Decreased MDA and NO levels, increased CAT, SOD, and GPx	Goudarzi et al. (2019)
Phthalates	500 mg/kg, daily for 4 weeks, intraperitoneal	2 mg/kg, daily for 4 weeks, oral	Male Wistar rats	Increased SOD, CAT, GPx activities and GSH level	Başak Türkmen et al. (2022)
Malathion	0.5 and 1 mg/L, 14 days, oral	100 mg/kg, 14 days, oral	Cyprinus carpio fish	Reduced SOD and CAT	Ural et al. (2015)
Polychlorinated biphenyl	2 mg/kg, daily for 8 weeks, intraperitoneal	2 mg/kg, every other day for 8 weeks, oral	Male Sprague–Dawley rats	Suppressed lipid peroxidation, enhanced GSH level, and GSH‐Px and CAT activities	Ateşşahin et al. (2010)
Nicotine	0.125, 0.25, 0.5, 1, 2, 3 and 4 mM, 1 h	10, 50, 100, 150, and 300 μM, 1 h	Wistar rats lymphocytes	Increased SOD, CAT, and GPx activities	Sudheer et al. (2007)
Diazinon	200 mg/kg, 24 h, oral	85 mg/kg, 24 h, oral	Female Sprague–Dawley rats.	Increased AChE activity and decreased GGT and amylase activities	Alp et al. (2011)
Endrin	4.5 mg/kg, 24 h, oral	6.0 mg/kg, 3 days, oral	Female Sprague–Dawley rats	Decreased lipid peroxidation, MDA, acetaldehyde, formaldehyde, acetone and DNA single‐strand breaks	Bagchi et al. (1993)

Abbreviations: AChE, acetyl cholinesterase; ALT, alanine transaminase; AST, aspartate transaminase; CAT, catalase; GGT, γ‐glutamyltransferase; GPx, glutathione peroxidase; GSH, glutathione; GSH‐Px, plasma glutathione peroxidase; HO‐1, heme oxygenase‐1; HP, hydroperoxides; IL‐1β, interleukine‐1β; IL‐6, interleukine‐6; MDA, malondialdehyde; NF‐κB, nuclear factor‐kappa‐B; NO, nitric oxide; NQO1, quinone oxidoreductase 1; Nrf2, nuclear factor erythroid‐2‐related factor 2; RB, retinoblastoma; SOD, superoxide dismutase; SR‐B1, scavenger receptor class B type 1; TAS, total antioxidant status; TBARS, thiobarbituriacid reactive substances; TGF‐β1, transforming growth factor‐β1; TNF‐α, tumor necrosis factor‐alpha; TOS, total oxidant status.

### 5.1 Paraquat

Paraquat (1,1′‐dimethyl‐4,4′ bipyridinium dichloride) is a toxic and inexpensive bipyridylium herbicide that is widely used. Paraquat is fatal in very small amounts and provokes toxic effects on many organs such as the lungs, liver, kidney, heart, stomach, and intestine (Amin et al., 2021). In an animal model, EA showed a protective effect against paraquat‐induced kidney dysfunction by increasing the total antioxidant status (TAS) and decreasing the total oxidant status (TOS) (Table 3; Silfeler et al., 2017). Thus, it could be concluded that EA, due to its antioxidant properties, might block oxidative injury and kidney toxicity induced by paraquat.

### 5.2 Ethanol

Ethanol's toxicity is divided into acute and chronic. Acute alcohol intoxication has well‐known clinical manifestations, including neuropsychiatric symptoms, intellectual excitation, intellectual and psychic excitation, cerebellar syndrome accompanying marked drunkenness, and deep coma (Le Daré et al., 2019). Chronic ethanol exposure is toxic to several organs such as digestive tract steatosis, hepatic cirrhosis, pancreatitis, cerebellar atrophy, polyneuritis, chronic gastritis, memory, and cardiovascular dysfunction (Le Daré et al., 2019). Different pathways play a role in ethanol‐induced tissue injury such as the formation of 1‐hydroxyethyl radicals, changes in cellular NAD^+^/NADH, and ethanol‐mediated mitochondrial damage (Gopal et al., 2021; Li et al., 2022). Sohn and colleagues showed that the protective effect of EA on alcohol‐induced toxicity was induced by regulating NO, scavenger receptor class B type 1 (SR‐B1), and transforming growth factor‐β1 (TGF‐β1) production in human hepatocellular carcinoma (HepG2) cells. Treatment of hepatic HepG2 cells with EA significantly decreased TGF‐β1 and NO expression. SR‐B1 plays a significant role in mediating the uptake of cholesteryl ester and high‐density lipoprotein (HDL)‐derived cholesterol in the liver. In liver cells, EA has dose‐dependent differential effects on SR‐B1 expression regulation. At 100 μM, EA elevated SR‐B1 expression in ethanol‐treated cells (Table 3; Sohn et al., 2013). Another study indicated that EA supplementation fed with ethanol for 45 days reduced AST, ALT, thiobarbituric acid reactive substances (TBARS), and hydroperoxides (HP), as well as modulated lipid metabolism. The study also showed a marked histopathological amelioration of kidney toxicity following the EA treatments (Devipriya et al., 2008).

### 5.3 Phosalone

Phosalone (6‐chloro‐3‐[diethoxyphosphinothioylsulfanylmethyl]‐1,3‐benzoxazol‐2‐one) is one of the most common organophosphorus pesticides (Ghasemi‐Niri et al., 2016). Phosalone stimulates inflammatory factors including TNF‐α and NF‐κB and subsequently initiates p53 induction. It also accelerates the oxidative pathway by up‐regulation of p38, p53, and retinoblastoma (RB) genes (Altuntas et al., 2003). EA exerted protective effects against phosalone‐induced senescence and decreased inflammatory markers such as TNF‐α, IL‐1β, and IL‐6β. EA down‐regulated NF‐κB, RB, and p53 at all concentrations. Analysis of the cell cycle (G0/G1, S, and G2/M phases) showed that treatment of phosalone‐induced cells at all concentrations with EA remarkably elevated the ratio of cells in the S phase compared to the phosalone group. It was found that EA progressively deactivated the phosalone‐induced senescence in REF cells, dose‐dependently (Table 3; Baeeri et al., 2017).

### 5.4 Rotenone

Rotenone is an isoflavone classified as “moderately hazardous.” The plant‐derived pesticide rotenone is a powerful complex I‐specific inhibitor that is generally applied to model optic neuropathies and Parkinson's disease in the lab (Cimdins et al., 2019). Wei et al. showed that EA protected dopamine neurons from rotenone‐induced neurotoxicity by activating the nuclear factor erythroid‐2‐related factor 2 (Nrf2) signaling pathway. They showed that EA treatment improved locomotor dysfunction in rotenone‐treated mice. EA induced higher Nrf2, quinone oxidoreductase 1 (NQO1), and heme oxygenase‐1 (HO‐1) expressions (Table 3; Wei et al., 2020). In brief, EA by stopping oxidative stress and activating the Nrf2 signaling pathway protected dopamine neurons from rotenone‐induced neurotoxicity.

### 5.5 Acrylamide

Acrylamide (C_3_H_5_NO) is a food contaminant present in a vast range of frequently consumed foods, which makes people's exposure to this toxicant unfortunately unavoidable. Acrylamide promotes neurotoxicity, mutagenicity, and carcinogenicity (Rifai & Saleh, 2020). Acrylamide exposure increases ROS, MDA, and inflammatory cytokines, for example, TNF‐α and IL‐6 levels while decreasing GSH content. In a rat model of acrylamide‐induced neurotoxicity, animals were pretreated with EA. They showed lower MDA and NO levels. Also, EA intensified the endogenous antioxidant defense system by enhancement of CAT, SOD, and GPx activities (Table 3; Goudarzi et al., 2019).

### 5.6 Phthalates

Phthalates are synthetic chemicals with colorless, poorly water‐soluble, and low volatility properties. Phthalates are widely used in the plastic industry. Humans are continuously exposed to phthalates through different routes of exposure such as ingestion, inhalation, or dermal absorption (Lyche et al., 2009). Several studies indicated that phthalates cause diverse toxicities in humans and animals, such as neurotoxicity, genotoxicity, hepatotoxicity, and immunotoxicity mainly via oxidative stress induction. It was shown that EA prevented phthalates toxicities by an increase in SOD, CAT, and GPx activities, and GSH enhancement in rats. The study also indicated that EA significantly increased sperm concentration, seminiferous tubular diameter (MSTD), germinal epithelium thickness (GECT), and decreased abnormal sperm rate (Table 3; Başak Türkmen et al., 2022).

### 5.7 Malathion

Malathion (*O*,*O*‐dimethyl‐*S*‐1,2‐bis ethoxy carbonyl ethyl phosphorodithioate) is an organophosphate insecticide that is commonly used to control mosquitoes and various insects that attack vegetables, fruits, shrubs, and landscaping plants (Badr, 2020). In slight oxidative stress, due to compensatory response, the activities of antioxidant enzymes such as SOD, CAT, GSH‐Px, and GST are increased while severe oxidative stress depresses the activities of these enzymes due to oxidative injury and a loss in compensatory mechanisms (Zhang et al., 2004). In accordance, malathion caused notable enhancement in the tissue CAT and SOD activities. However, treatment with EA reduced SOD and CAT activities following malathion exposure which may be due to its hydrogen peroxide scavenging and superoxide anion radical scavenging properties (Table 3; Ural et al., 2015).

### 5.8 Polychlorinated biphenyl

In recent years, industrial and agricultural development has led to an increase in environmental pollutants. Polychlorinated biphenyls are a group of extensively spread environmental pollutants that disrupt normal endocrine functions in animals and humans (Abraham et al., 2002). EA protects against polychlorinated biphenyl‐induced inflammation by suppressing lipid peroxidation and enhancing antioxidant enzyme activities such as SOD, and CAT. EA administration to polychlorinated biphenyl‐treated animals remarkably prevented the reduction in diameters of seminiferous tubules, Johnsen's testicular score, and germinal cell layer thickness (Table 3; Ateşşahin et al., 2010).

### 5.9 Nicotine

It is presumed that the toxic effects of nicotine are partly due to the raised production of ROS. ROS causes oxidative injury to several molecules in cells, proteins, membrane lipids, and nucleic acids (Hajam et al., 2022). EA efficiently decreased TBARS and HP, increased SOD, CAT, and GPx activities, and improved nicotine‐induced DNA damage (Table 3; Sudheer et al., 2007).

### 5.10 Diazinon

Diazinon (*O*,*O*‐diethyl‐*O*‐(2‐isopropyl‐4‐methyl‐6‐pyrimidinylphosphorothionate) is a common organophosphate pesticide to control insects in household environments and agriculture. Although it has a low persistence in the natural world, it is a general insecticide that is very toxic to humans and animals (Shah & Iqbal, 2010). Diazinon reduced acetylcholinesterase (AChE) activity and raised amylase and GGT activities. It also disrupted the liver, kidney, and lung tissues. The adverse effects of diazinon on these enzymes were established histopathologically (Alp et al., 2011). EA could block the toxic effects of diazinon that were seen as negative modifications of the serum amylase, GGT, and AChE, in concomitant with damages to the liver, lungs, and kidneys. Histopathologic outcomes revealed that this compound partially prevented and decreased the degenerative toxic effects of diazinon (Table 3; Alp et al., 2011).

### 5.11 Endrin

Endrin (1,2,3,4,10,10‐hexachloro‐6,7‐epoxy‐1,4,40t,5,6,7,8,8a‐octahydroendo,endo‐l,4:5,8 dimethanonaphthalene) is a very toxic chlorinated cyclodiene insecticide having a larger spectrum of activity than other cyclodienes for example aldrin and dieldrin. Several biochemical changes are caused by endrin's toxicity (Matsumoto et al., 2009). Rats exposed to endrin showed raised lipid peroxidation and DNA single‐strand breaks (DNA‐SSB), whereas EA treatment reversed these changes. EA pretreatment prior to endrin administration reduced acetaldehyde, MDA, formaldehyde, and acetone by 90%, 86%, 87%, and 73%, respectively. Consequently, the results demonstrated that EA attenuated the biochemical abnormalities of endrin‐induced oxidative stress (Table 3; Bagchi et al., 1993).

## 6 CONCLUSION

In conclusion, based on the evidence provided in the literature, EA has significant therapeutic effects against non‐natural and natural toxins. These effects are mainly mediated through EA's antioxidant, anticancer, and antiinflammatory properties. In vivo and in vitro models revealed various mechanisms are responsible for the protective effects of EA. Selected mechanisms include up‐regulation of Nrf2 and GSH, an increase in CAT, SOD, and GPx activities, and down‐regulation of NF‐κB expression. Furthermore, EA's anti‐apoptotic potential was indicated to be mediated by regulating Bax, Bcl‐2, and caspase3/9 and phosphorylation of ERK1/2. Although a significant body of evidence regarding the protective properties of EA against various chemical and natural toxins exists, clinical trials are necessary before reaching a certain result on the compound's protective potential against diverse toxic compounds. Additionally, the pharmacodynamics/pharmacokinetics of EA should be investigated comprehensively to provide a more clear picture of its proper dose, bioavailability, tolerability, and efficacy.

## AUTHOR CONTRIBUTIONS

**Zahra Jamshidi:** Data curation (equal); writing – original draft (equal). **Ali Roohbakhsh:** Writing – review and editing (equal). **Gholamreza Karimi:** Conceptualization (equal); writing – review and editing (equal).

## CONFLICT OF INTEREST STATEMENT

The authors declare that they have no conflict of interest.

## Data Availability

Data are availabe as request.
